# Supplementary material for: “Doctor, when can I drive?” – Can we compensate an immobilization of the right wrist while driving a car: A pilot study
Source: PLoS One. 2026 Feb 18;21(2):e0341628. doi: 10.1371/journal.pone.0341628 (PMC12915907; doi:10.1371/journal.pone.0341628)
Supplement: Attachment — Submitted filename: reportPLOS.docx [file pone.0341628.s023.docx]

**Overall Assessment**

The manuscript addresses an interesting and clinically relevant question concerning the impact of wrist immobilization on driving ability. This is a pertinent topic in orthopedic and trauma care, as patients frequently inquire about their fitness to drive following immobilization or surgery. The use of a driving simulator combined with motion capture analysis is a clear strength and adds objectivity to the study. The paper is well-structured, the methodology is described in detail, and the results are generally clear. However, several methodological, analytical, and reporting issues require clarification and revision before the work can be considered for publication.

**Strengths**

1. **Novelty and Relevance**: The study focuses on an underexplored area—upper extremity immobilization and compensatory strategies during driving.
2. **Methodological Rigor**: Use of motion capture and a standardized driving simulator protocol provides objective kinematic data.
3. **Clinical Importance**: Results may aid physicians in counseling patients regarding return to driving after wrist immobilization.
4. **Clear Structure**: The manuscript follows a logical format with clear separation of Introduction, Methods, Results, and Discussion.

**Major Concerns**

1. **Sample Size and Power**
   - Only 20 participants were included, all of them healthy volunteers without actual injury or pain. While the study demonstrates biomechanical compensation, the small and homogeneous sample raises questions about generalizability.
   - A power analysis to justify the sample size is not provided.
2. **External Validity**
   - The study used healthy young adults (mean age ~28 years) without pain or disability. This limits applicability to real patients who often have pain, swelling, decreased grip strength, or comorbidities.
   - This limitation is acknowledged but not sufficiently emphasized in the Discussion. A stronger statement is needed about how this affects clinical translation.
3. **Simulator Limitations**
   - Only three driving scenarios were tested (shifting, left turn, right turn), which may not represent the complexity of real-life driving (e.g., emergency braking, rapid steering corrections, highway driving).
   - Automatic transmission was used in some parts, potentially underestimating the role of the immobilized wrist.
4. **Statistical Analysis**
   - The choice of linear mixed models is appropriate, but details of the model specification are sparse.
   - It is not clear if adjustments for multiple comparisons were performed, given the large number of joint angles and outcomes analyzed. This raises the risk of type I errors.
   - Effect sizes and confidence intervals are not presented, which would strengthen the interpretation.
5. **Clinical Implications**
   - The conclusion that “safe driving with the right immobilized wrist is possible” may be overstated given the limited scenarios and lack of patient population.
   - A more cautious interpretation is needed, highlighting that these findings apply only to healthy individuals in controlled conditions.

**Minor Concerns**

1. **Abstract**
   - The abstract is generally well written but should explicitly state that only healthy volunteers were studied.
   - Reporting of key statistical values (p-values, confidence intervals) would strengthen clarity.
2. **Introduction**
   - While background references are provided, the review of existing literature could be more concise. Some citations appear repetitive.
   - Clarify how this study advances beyond earlier work on elbow immobilization and driving.
3. **Methods**
   - More information about randomization (order of immobilized vs. non-immobilized trials) is needed.
   - Describe the wrist immobilization device more clearly—was thumb immobilization included? How standardized was the splint?
   - Provide explicit exclusion criteria (e.g., musculoskeletal disorders, neurological conditions).
4. **Results**
   - Tables are informative but could be simplified; some parameters (e.g., minimum/maximum values) may not add substantial value.
   - Figures would benefit from clearer legends and consistent labeling (NI = no immobilization; WO = with immobilization).
5. **Discussion**
   - The discussion is comprehensive but occasionally repetitive.
   - Greater emphasis on the limitations (young healthy participants, lack of pain, limited scenarios) is necessary.
   - Some references are dated; ensure that the most recent literature (past 5 years) is adequately included.
6. **Language and Style**
   - Overall, the manuscript is well written. Minor grammatical edits and simplification of long sentences would improve readability.

**Suggestions for Improvement**

- Include a **power analysis** or at least a rationale for the sample size.
- Report **effect sizes and confidence intervals** along with p-values.
- Clarify whether correction for multiple comparisons was applied.
- Strengthen the **limitations section**, particularly regarding generalizability to real patients.
- Revise the **conclusion** to be more cautious, stressing that results cannot be directly generalized to injured or post-operative populations.
- Improve clarity of tables and figures, and ensure consistent terminology.

**Conclusion and Recommendation**

This is a **well-designed pilot study** that provides valuable biomechanical insights into compensatory mechanisms during driving with wrist immobilization. However, methodological clarifications, more cautious interpretation, and improvements in reporting are needed.
