# Supplementary material for: “Doctor, when can I drive?” – Can we compensate an immobilization of the right wrist while driving a car: A pilot study
Source: PLoS One. 2026 Feb 18;21(2):e0341628. doi: 10.1371/journal.pone.0341628 (PMC12915907; doi:10.1371/journal.pone.0341628)
Supplement: Attachment — Submitted filename: Response to Reviewers.docx [file pone.0341628.s025.docx]

Dear academic editor and reviewers,

Thank you for reviewing our manuscript and for your valuable, constructive suggestions for improvement.

**Academic Editor**

We address your comments below:

1) We have revised the manuscript regarding the style requirements. If further adjustments are necessary, we will be happy to make them upon specific notification.

2) We would like to make the data underlying our study available to other researchers as supplementary information.

3) The ORCID ID of the corresponding author, Felix Lakomek, is: 0009-0000-1074-0246.

4) This point does not apply to our manuscript and was therefore not considered.

**Reviewer #1:**

We have added a description of the motion capturing system (Xsens).

**Reviewer #2:**

As requested, we simplified the sentence about the study's objective in the abstract and expanded the information in the results section.

We also addressed the sample size and, as you suggested, explained that this is an exploratory study. We described the procedure for randomizing in more detail and added your points about the study's limitations.

**Reviewer #3:**

First, we would like to address your most important concerns:

1. Sample size and power

We decided to include healthy volunteers to achieve the best possible comparability in basic research. This excluded influencing factors such as reduced responsiveness, chronic mobility restrictions, and visual impairment. Furthermore, we selected volunteers who were pain-free, as it is difficult to assess the extent to which pain affects each individual volunteer.

A power analysis was not performed prior to the study because it’s an exploratory study and no comparable studies were available.

Therefore, we based the sample size on the basic research studies conducted by our research group (*Latz, D; Schiffner, E; Schneppendahl, J, et al.: Doctor, when can I drive? -Range of functional ankle motion during driving. Foot and ankle surgery: official journal of the European Society of Foot and Ankle Surgeons. 10.1016/j.fas.2019.12.006; Latz, D; Schiffner, E; Schneppendahl, J, et al.: Doctor, when can I drive? - the range of elbow motion while driving a car. J Shoulder Elbow Surg. 28: 1139-1145. 10.1016/j.jse.2018.11.053; Latz, D; Schiffner, E; Schneppendahl, J, et al.: Doctor, when can I drive? - Range of motion of the knee while driving a car. The Knee. 26: 33-39.10.1016/j.knee.2018.11.005; Latz, D; Pfau, S; Koukos, C, et al.: Doctor, when can I drive. Obere Extremität. 12: 234-241. 10.1007/s11678-017-0426-0*).

2. External Validity

You are right about the limited applicability to real patients. Therefore, we have adjusted the limitations and conclusion sections.

3. Limitations of the Simulator

The volunteers completed a scenario that required shifting gears and making left and right turns. These are typical maneuvers required when driving a car. Further investigation of the maneuvers you mentioned, such as rapid steering corrections, is of clinical relevance. However, the aim of this study was not to make a statement about reaction time and the lower extremities.

During cornering, only an automatic transmission was initially used in basic research. When to shift gears while cornering is an individual decision of the driver, which would lead to poor comparability. As you noted, this can lead to the role of the immobilized right wrist being underestimated. In the conclusion, we explicitly refer to the critical combination of shifting gears and simultaneous steering.

4. Statistical Analysis

No adjustment for multiple comparisons was performed. This is now mentioned under limitations.

The 95% confidence intervals for difference were added as requested. Reporting effect sizes is very useful from a statistical perspective, as you already mentioned.

Since our analysis was conducted with SPSS, and SPSS does not allow the calculation of effect sizes for the models used, the effect size could not be calculated.

5. Clinical Implications

The conclusion has been adjusted to include a more cautious interpretation of our results under controlled conditions in healthy individuals.

We now address your minor concerns:

1. Summary

The summary now explicitly states that only healthy volunteers were included. Furthermore, the 95% confidence intervals for difference have been added.

2. Introduction

This study differs from previous work on wrist immobilization by combining a driving simulator with a motion capture system. A comparable study on the elbow joint has already been published by our research group (*Schiffner E, Lakomek F, Hilsmann F, Schoeps D, Prost M, Beyersdorf C, Windolf J, Latz D. Doctor, when can I drive? - compensation capability while driving with restricted elbow - a biomechanical analysis. JSES Int. 2024 Nov 1;9(2):542-548. doi: 10.1016/j.jseint.2024.09.028. PMID: 40182246; PMCID: PMC11962544*). This was clarified again in the introduction.

3. Methods

The order of the respective rides with the right wrist immobilized and the right wrist not immobilized was randomized using a random generator. The ride without immobilization was coded as 0, and the ride with the right wrist immobilized was coded as 1. The order was randomized individually for each volunteer.

The right wrist was immobilized using a palmar hard cast splint without thumb inclusion.

The following exclusion criteria were chosen musculoskeletal disorders, neurological disorders, active drug use, and lack of driving experience.

4. Results

In our opinion, the tables have already been reduced to a minimum; further simplification would, in our opinion, result in a loss of information. For example, in Table 1, the parameter "minimum" was omitted for speed.

The legends and labels of the tables and figures have been adjusted.

5. Discussion

A stronger emphasis on the limitations has been made as requested.

A recent literature review was conducted to compile this discussion, and we believe the literature is comprehensive regarding the topic being discussed.

6. Language and Style

Changes have been made to improve readability.

We hope that the implementation of your constructive criticism has led to a further improvement in the quality of the manuscript. Thank you very much.

With kind regards,

Falk Hilsmann
