# Supplementary material for: “Doctor, when can I drive?” – Can we compensate an immobilization of the right wrist while driving a car: A pilot study
Source: PLoS One. 2026 Feb 18;21(2):e0341628. doi: 10.1371/journal.pone.0341628 (PMC12915907; doi:10.1371/journal.pone.0341628)
Supplement: Attachment — Submitted filename: Response_to_Reviewers_auresp_2.docx [file pone.0341628.s026.docx]

Dear Sir or Madam,

Thank you for reviewing our revised manuscript. We are pleased to submit a revised version of our manuscript in accordance with your points of criticism, to meet the required publication criteria.

We will now briefly address the points of criticism you raised:

**Journal requirements:**

As mentioned in our previous letter, there was no specific recommendation regarding the citation of particular, previously published works. Therefore, no review for relevance and citability was conducted.

Furthermore, the bibliography was reviewed again for completeness and accuracy. No irregularities were found; in particular, no retracted works are cited.

**Additional comments from the editors:**

The manuscript has been revised as requested, including an adjustment of the title and other necessary changes.

We now hope that the manuscript will be accepted as a pilot study.

Thank you in advance.

Sincerely,

Falk Hilsmann
